# Depression in childhood to early adulthood and respiratory health in early adulthood

**DOI:** 10.1192/bjo.2024.794

**Published:** 2024-11-11

**Authors:** Gang Wang, Jenny Hallberg, Natalia Hernandez-Pacheco, Sandra Ekström, Ellen Vercalsteren, Bronwyn K. Brew, Catarina Almqvist, Christer Janson, Inger Kull, Anna Bergström, Erik Melén, Donghao Lu

**Affiliations:** Division of Internal Medicine, Institute of Integrated Traditional Chinese and Western Medicine, West China Hospital, Sichuan University, Chengdu, China; Department of Clinical Science and Education, Södersjukhuset, Karolinska Institutet, Stockholm, Sweden; Institute of Environmental Medicine, Karolinska Institutet, Stockholm, Sweden; Health Management Center, General Practice Medical Center, Medical Device Regulatory Research and Evaluation Center, West China Hospital, Sichuan University, Chengdu, China; Sachs’ Children and Youth Hospital, Södersjukhuset, Stockholm, Sweden; CIBER de Enfermedades Respiratorias (CIBERES), Instituto de Salud Carlos III, Madrid, Spain; Respiratory, Allergy and Sleep Research, Department of Medical Sciences, Uppsala University, Uppsala, Sweden; Department of Medical Epidemiology and Biostatistics, Karolinska Institutet, Solna, Sweden; National Perinatal Epidemiology and Statistics Unit, Centre for Big Data Research in Health and School of Clinical Medicine, University of New South Wales, Kensington, New South Wales, Australia; Pediatric Allergy and Pulmonology Unit, Astrid Lindgren Children's Hospital, Karolinska University Hospital, Stockholm, Sweden; Centre for Occupational and Environmental Medicine, Region Stockholm, Stockholm, Sweden

**Keywords:** Adolescents, early life factors, inflammation biomarker, mediation analysis, smoking

## Abstract

**Background:**

Both depression and respiratory disease are common today in young populations. However, little is known about the relationship between them.

**Aims:**

This study aims to explore the association between depression in childhood to early adulthood and respiratory health outcomes in early adulthood, and the potential underlying mechanisms.

**Method:**

A prospective study was conducted based on the Swedish BAMSE (Barn, Allergi, Miljö, Stockholm, Epidemiologi [Children, Allergy, Milieu, Stockholm, Epidemiology]) birth cohort (*n* = 4089). We identified clinically diagnosed depression through the dispensation of antidepressants, using national register data confirmed by self-reported diagnosis. At the 24-year follow-up, respiratory health was assessed via questionnaires and clinical evaluation. Metabolic and inflammatory profiles were analysed to explore potential mechanisms.

**Results:**

Among the 2994 participants who provided study data, 403 (13.5%) had depression at any time point from around age 10 to 25 years. Depression was associated with higher risks of any chronic bronchitis symptoms (odds ratio = 1.58, 95% CI 1.21–2.06) and respiratory symptoms (odds ratio = 1.41, 95% CI 1.11–1.80) in early adulthood, independent of body mass index (BMI) and smoking status. Compared to individuals without depression, those with depression had a higher fat mass index (FMI (β = 0.48, 95% CI 0.22–0.74)) and increased blood levels of fibroblast growth factor 21 and Interleukin-6 in early adulthood. These markers together with FMI were found to partly mediate the association between depression and respiratory symptoms (total mediation proportion: 19.8 and 15.4%, respectively, *P* < 0.01).

**Conclusions:**

Depression in childhood to early adulthood was associated with an increased risk of respiratory ill-health in early adulthood, independently of smoking. Metabolic and inflammatory dysregulations may underlie this link.

In adolescents and young adults, depressive disorders are the most common mental illness, and its prevalence increased sharply in the past decade.^1^ Moreover, depression disorders are ranked the fourth leading cause of disease burden out of all health conditions among adolescents and young adults.^2^ During childhood to early adulthood, the incidence of depression sharply increases,^3^ with around 40% of the patients experiencing their first episode of depression before 20 years of age.^1,4^ Moreover, depression in adolescents and young adults has been associated with a range of negative health outcomes later in life, such as cardiovascular disease^5^ and premature death.^6^ In addition, emerging data suggest that depression is linked to developing metabolic syndrome, pro-inflammation profiles and unhealthy behaviour (such as cigarette smoking), which may lead to negative respiratory health outcomes in adolescents and young adults.

However, less is known about the association between childhood/adolescent depression and later respiratory health. Respiratory symptoms, such as breathlessness, chest tightness, wheezing, mucus production and coughing, are common,^7,8^ and associated with lowered quality of life and worse health outcomes in adolescents and young adults. Shared genetic factors between asthma and depression and anxiety have been reported in children, adolescents and adults.^9,10^ Besides, previous studies have shown that adulthood depression is associated with respiratory symptoms,^11–14^ but not with asthma or bronchial responsiveness.^11^ However, no study has explored the potential mechanisms that underlie the association between depression and respiratory health outcomes using biological markers. As systemic inflammation and metabolic dysfunction are well known targetable factors which relate to both depression^15,16^ and respiratory health outcomes,^17,18^ they are worth being explored as the potential underlying factors linking the association. Thus, understanding the potential biological mechanisms, for instance, through metabolic and/or inflammatory pathways, may provide important evidence for developing strategies for the early detection and intervention of depression-associated respiratory symptoms and diseases in adolescents and young adults.

Therefore, we conducted a prospective study, examining the association between depression in childhood to early adulthood and respiratory outcomes in early adulthood, and exploring the potential underlying mechanisms of the association through metabolic and inflammatory biomarkers.

## Methods

### Study population and ethics

The Swedish population-based birth cohort BAMSE (Barn, Allergi, Miljö, Stockholm, Epidemiologi [Children, Allergy, Milieu, Stockholm, Epidemiology]) involved 4089 infants from inner-city, urban and suburban districts of Stockholm (Sweden) between February 1994 and November 1996 and followed them from birth through 24 years.^19,20^ The BAMSE project invited all the newborn children in the study areas, and people were excluded according to the following criteria: (a) the family planned to move within 1 year of the study start, (b) insufficient knowledge of the Swedish language, (c) the family had a seriously ill child (d) or an older sister or brother was already included in the study. The study was approved by the Regional Ethical Review Board in Stockholm (ref. 2016/1380-31/2). The parents (at inclusion and at participants’ ages 4 and 8 years) and participants (at age 16 and 24 years) signed their informed consent, under the Helsinki Declaration.

### Follow-up from childhood to young adulthood

Additional study details are presented in the online Supplementary material, some of which have been described elsewhere.^20,21^ Briefly, information on demographics, lifestyle and health conditions (e.g. childhood asthma and respiratory infections) was obtained from parental questionnaires administered at recruitment and follow-up visits when the children were aged 1, 2, 4, 8, 12 and 16 years. The participants answered the questionnaire themselves at the age of 16 and 24 years.^22^ The 24-year follow-up started when the first wave of participants reached 24 years of age.

### Assessment of depression

Every resident in Sweden is assigned a unique identification number, which is linked to the Prescribed Drug Register, which records all filled dispensations since July 2005 in any pharmacy in Sweden.^23^ All dispensations of antidepressants, including selective serotonin reuptake inhibitors (anatomical therapeutic chemical code N06AB), non-selective serotonin reuptake inhibitors (N06AA) and other antidepressants (N06A), during 2005–2019 (i.e. ages 10–25 years) were identified.

In addition, at the 24-year follow-up, participants were asked in a survey: ‘Do you have or have you previously had depression?’. Because antidepressants could be prescribed for disorders other than depression, depression was defined as both a record of antidepressant dispensation and self-confirmed depression. Participants with no record of antidepressants and no self-confirmed depression were classified as having no depression. Participants with only a record of antidepressants or self-confirmed depression were excluded from the main analysis but included in the sensitivity analysis.

### Assessment of respiratory health outcomes at the 24-year follow-up

We assessed a range of respiratory conditions via questionnaires. Respiratory symptoms were assessed as any troublesome breathing, chest tightness or wheezing during the last 12 months. Chronic bronchitis was defined as the combination of the symptoms of cough and mucus production in the morning during winter.^20^ Current asthma was defined as fulfilling at least two of the following three criteria: (a) positive answer to a doctor's diagnosis of asthma, (b) wheezing in the past 12 months or (c) use of asthma medication during the past 12 months.

Moreover, we evaluated respiratory conditions using objective exams. Lung function testing was performed at the 24-year follow-up using the Vyaire Vyntus system (Vyaire Medical, Chicago, IL, USA).^24^ Pre- and post-bronchodilator spirometry measures were determined following the American Thoracic Society/ European Respiratory Society (ATS/ERS) recommendations. For each lung function test, the highest values of forced expiratory volume in 1 s (FEV_1_) and forced vital capacity (FVC) were used for analysis. Predicted values and *z* scores of FEV_1_ and FVC were calculated from the Global Lung Function Initiative (GLI) equations.^25^ Fractional exhaled nitric oxide (FeNO) was measured at the 24-year follow-up for potential airway inflammation using the NIOX vero analyzer (Aerocrine AB, Solna, Sweden) according to the ATS/ERS guidelines. Airborne and food allergen sensitisation was assessed: a mix of common airborne allergens were tested for with Phadiatop®, and a combination of common food allergens with fx5® (ImmunoCAP System; ThermoFisher, Uppsala, Sweden). A positive test was defined as allergen-specific immunoglobulin E (IgE) ≥0.35 kilounits of allergen-specific IgE per litre (kUA/L). Data on eosinophil and neutrophil counts were available from clinical follow-up.

### Metabolic and inflammatory profiles

To shed light on potential biological mechanisms, we obtained data on metabolic and inflammatory profiles. Venous blood was collected at the 24-year clinical follow-up. The expression levels of 92 proteins (details of the proteins can be found in Supplementary Table 1, available at https://doi.org/10.1192/bjo.2024.794) in plasma were analysed by using the Proseek Multiplex Inflammation Panel (Olink Biosciences, Uppsala, Sweden). Details of the proteomics analysis have been described previously.^26^ Data are presented as Normalised Protein eXpression (NPX) values, which are Olink Proteomics’ arbitrary units on a log_2_ scale.

Data on height, weight and blood lipids were available from the 24-year clinical follow-up. Body mass index (BMI) was calculated as weight in kilograms divided by height in meters squared (kg/m^2^) and then divided into four categories: underweight (<18.5 kg/m^2^), normal weight (18.5–24.9 kg/m^2^), overweight (25–29.9 kg/m^2^) and obese (>30 kg/m^2^).^27^ Bioimpedance measurements were tested at the 24-year follow-up using the Tanita MC 780 body composition monitor based on the instructions from the manufacturer. Trunk fat, body fat and fat-free mass were included in the current analysis. Fat mass index (FMI) and fat-free mass index were calculated as fat mass and fat-free mass in kg/m^2^.

### Statistical analyses

Covariates were compared between participants with or without depression using the *t*-test/analysis of variance (ANOVA) test, Kruskal–Wallis rank sum test and Chi-squared test as appropriate. Associations between depression and respiratory health-related outcomes at the 24-year follow-up were investigated using multivariable logistic or linear regression models as appropriate, adjusted for demographics (age, gender and parental education level) in model 1, and BMI and current smoking status at the 24-year follow-up in model 2. The associations between depression and metabolic profiles were also explored using those two models excluding BMI in model 2.

Protein levels were normalised based on inverse normal transformation.^28^ Association between depression and each protein level was investigated using linear regression models adjusted for age, gender, parental education level, BMI and current smoking status at the 24-year follow-up. Multiple comparisons were corrected by applying the Benjamini–Hochberg method.

The potential mediation^29^ by inflammatory and metabolic profiles on the depression and respiratory symptom associations were estimated using Bayesian regression models and adjusting for potential covariates.^30^ The serial mediating effects of several mediators on the relationship were also explored.^29,30^ The top inflammatory and metabolic markers were selected as the meditators.

Early-life factors (such as premature birth, maternal smoking during pregnancy, birth weight, exclusive breastfeeding for more than 4 months, bronchitis during 0–1 years, pneumonia during 0–1 years, respiratory syncytial virus infections during 0–1 years and pneumonia during 0–4 years) were explored as potential confounders and further adjusted in the sensitivity analysis. Additionally, association analyses were also conducted in participants who were dispensed with antidepressant medication at younger than 18 years of age to test the potential effect of reverse causation, and in participants with a wider definition (either a filled dispensation of antidepressants or self-reported depression) of depression to test the robustness of the results.

All the analyses were performed using R, version 4.1.2, in Windows.

## Results

### Baseline characteristics

Among the 4089 children originally included in the BAMSE cohort, 3064 (75%) participants completed the questionnaire at the 24-year follow-up (Supplementary Fig. 1). Of these respondents, 2994 (97.7%; 1586 females) provided information on depression. Compared with people who were not included, participants included in the current study were more likely to be female and have higher parental education levels. Additionally, there was less maternal smoking during pregnancy, more breastfeeding and less preschool wheezing in the participants included (Supplementary Table 2). The overall prevalence of depression up to the 24-year follow-up was 13.5% (*n* = 403). The median age at the first filled dispensation of antidepressants was approximately 19 years (ranging from 10 to 25 years). The most commonly used antidepressants were selective serotonin reuptake inhibitors (95.8%). There were more females in the depression group compared with the unaffected group (61.3% *v.* 50.3%, Table 1). Compared to participants without depression, those with depression had a higher prevalence of being overweight and having obesity at the 24-year follow-up. Moreover, there was more active smoking (29.0% *v.* 18.1%) in participants with depression. No statistically significant differences were found for depression versus no-depression in relation to age, second-hand smoking at the 24-year follow-up and parental education levels.

**Table 1:** Characteristics of BAMSE participants by depression

	No depression (*n* = 2150)	Depression (*n* = 403)	*P* values
Age at the first dispensations of antidepressants in years, median [range]	N/A	19.3 [10.2–25.0]	N/A
Age at the 24-year follow-up, years	22.49 ± 0.55	22.48 ± 0.50	0.60
Sex			<0.001
Female	1081 (50.3%)	247 (61.3%)	
Male	1069 (49.7%)	156 (38.7%)	
BMI at the 24-year follow-up, kg/m^2^	23.21 ± 3.60	23.54 ± 4.37	0.098
BMI categories at the 24-year follow-up^a^			0.010
Obesity	105 (4.9%)	33 (8.3%)	
Overweight	392 (18.3%)	79 (19.8%)	
Normal weight	1537 (71.9%)	261 (65.4%)	
Underweight	103 (4.8%)	26 (6.5%)	
Highest parental education			0.71
University	1208 (56.2%)	222 (55.2%)	
Primary school/high school	941 (43.8%)	180 (44.8%)	
Ever prescribed antidepressants			
SSRIs	N/A	386 (95.8%)	N/A
NSMRIs	N/A	9 (2.2%)	N/A
Other	N/A	139 (34.5%)	N/A
Smoking exposures at 24-year follow-up			
Current smoking	389 (18.1%)	117 (29.0%)	<0.001
Second-hand smoking	52 (2.6%)	10 (2.6%)	1.00

BAMSE, Barn, Allergi, Miljö, Stockholm, Epidemiologi [Children, Allergy, Milieu, Stockholm, Epidemiology]; BMI, body mass index; SSRIs, selective serotonin reuptake inhibitors; NSMRIs, non-selective serotonin reuptake inhibitors; N/A, not available.

The data were presented as mean ± standard deviation or number (%).

a. Standard BMI categories of underweight (<18.5 kg/m^2^), normal weight (18.5–24.9 kg/m^2^), overweight (25–29.9 kg/m^2^) and obese (>30 kg/m^2^) were used.

### Respiratory health outcomes at the 24-year follow-up

Respiratory symptoms and lung function data for each group are presented in Table 2. In the depression group, 30.5% reported any respiratory symptoms in the past 12 months compared to 21.9% in the unaffected group. The odds ratio for any respiratory symptom was 1.53 (95% CI = 1.21–1.94), with adjustment for demographics (model 1). The association was slightly attenuated yet remained significant after further adjusting for BMI and current smoking status (odds ratio = 1.41, 95% CI = 1.11–1.80; model 2). Stratified analysis showed a stronger association in females than in males (*P* for interaction = 0.046), whereas comparable associations were found across BMI groups and regardless of asthma at the 24-year follow-up (Supplementary Table 3). In addition, a positive association was noted for wheezing (defined as wheezing on more than three occasions) (odds ratio = 0.85, 95% CI = 1.39–2.47, model 2; Table 2), but not for asthma. Moreover, depression was positively associated with chronic bronchitis symptoms overall (odds ratio = 0.58, 95% CI = 1.21–2.06), particularly with only the symptom of mucus (odds ratio = 1.69, 95% CI = 1.20–2.37). No significant associations were observed between depression and respiratory infections, pre- and post-bronchodilator lung function, FeNO, neutrophil, eosinophil levels in the blood or sensitisation to airborne and food allergens (Table 2).

**Table 2:** Associations between childhood to early adulthood depression and respiratory symptoms and function at the 24-year follow-up

	No depression (*n* = 2150)	Depression (*n* = 403)	Model 1, odds ratio/ β [95% CI]^a^	Model 2, odds ratio/ β [95% CI]^b^
Respiratory symptoms				
Any symptom	470 (21.9%)	123 (30.5%)	**1.53 [1.21, 1.94]**	**1.41 [1.11, 1.80]**
Wheezing > three times	232 (10.8%)	77 (19.1%)	**1.89 [1.42, 2.52]**	**1.85 [1.39, 2.47]**
Asthma	293 (13.6%)	70 (17.4%)	1.30 [0.97, 1.73]	1.24 [0.93, 1.67]
Respiratory infections				
One to three times	1341 (65.9%)	246 (63.4%)	1.05 [0.74, 1.48]	1.06 [0.75, 1.50]
More than four times	427 (21.0%)	96 (24.7%)	1.28 [0.87, 1.88]	1.27 [0.86, 1.89]
Chronic bronchitis symptoms				
Any chronic bronchitis symptoms	322 (15.9%)	94 (24.3%)	**1.72 [1.32, 2.23]**	**1.58 [1.21, 2.06]**
Only cough	57 (2.8%)	14 (3.6%)	1.42 [0.78, 2.60]	1.31 [0.71, 2.40]
Only mucus	174 (8.6%)	52 (13.4%)	**1.76 [1.26, 2.47]**	**1.69 [1.20, 2.37]**
Chronic bronchitis	91 (4.5%)	28 (7.2%)	**1.80 [1.15, 2.80]**	1.52 [0.96, 2.40]
Lung function				
Pre-bronchodilator spirometry (*z* score)				
FEV_1_	−0.26 ± 0.85	−0.26 ± 0.87	−0.01 [−0.13, 0.10]	−0.05 [−0.16, 0.06]
FVC	−0.05 ± 0.84	−0.01 ± 0.86	0.03 [−0.07, 0.14]	−0.02 [−0.12, 0.09]
FEV_1_/FVC ratio	−0.36 ± 0.89	−0.41 ± 0.92	−0.07 [−0.18, 0.05]	−0.04 [−0.15, 0.08]
Post-bronchodilator spirometry (*z* score)				
FEV_1_	0.01 ± 0.83	0.01 ± 0.85	−0.01 [−0.12, 0.10]	−0.04 [−0.15, 0.07]
FVC	−0.09 ± 0.85	−0.05 ± 0.86	0.03 [−0.08, 0.14]	−0.01 [−0.13, 0.10]
FEV_1_/FVC ratio	0.11 ± 0.83	0.06 ± 0.87	−0.06 [−0.17, 0.05]	−0.03 [−0.15, 0.08]
FeNO, neutrophils, eosinophils and IgE sensitisation				
FeNO, ppb, median [IQR]^c^	12 [9, 19]	11 [8, 18]	0.45 [−1.52, 2.43]	0.49 [−1.50, 2.48]
Neutrophils in blood (% total cell count), median [IQR]^c^	56.1 [50.0, 62.3]	55.4 [49.1, 61.4]	−0.43 [−1.56, 0.69]	−0.55 [−1.69, 0.59]
Eosinophils in blood (% total cell count), median [IQR]^c^	1.5 [0.0, 3.0]	1.5 [0.0, 3.0]	0.02 [−0.26, 0.31]	0.01 [−0.28, 0.30]
Airborne allergen sensitisation^d^	677 (43.1%)	117 (39.0%)	0.90 [0.69, 1.16]	0.89 [0.69, 1.16]
Food allergen sensitisation^e^	131 (8.3%)	27 (9.0%)	1.14 [0.74, 1.77]	1.12 [0.72, 1.74]

FEV_1_, forced expiratory volume in 1 s; FVC, forced vital capacity; FeNO, fractional exhaled nitric oxide; IgE, immunoglobulin E; ppb, parts per billion; IQR, interquartile range; kUA/L, kilounits of allergen-specific IgE per litre.

The data were presented as mean ± standard deviation, median [IQR] or number (%). Statistically significant values are highlighted using bold text.

a. The estimates were adjusted for age, gender and parental education level.

b. The estimates were adjusted for age, gender, parental education level, BMI and current smoking status.

c. Based on Kruskal–Wallis rank sum test.

d. Sensitisation to a mix of common airborne allergens with Phadiatop® (ImmunoCAP System; ThermoFisher, Uppsala, Sweden. A positive test was defined as specific IgE ≥0.35 kUA/L).

e. This refers to a sensitisation to a mix of common food allergens with fx5® (ImmunoCAP System; ThermoFisher, Uppsala, Sweden. A positive test was defined as specific IgE ≥0.35 kUA/L).

### Metabolic profile

Depression was associated with increased body fat, trunk fat, FMI and decreased fat-free mass at the 24-year follow-up (Table 3). Moreover, depression was associated with higher low-density lipoprotein (LDL) and LDL/high-density lipoprotein (HDL) ratio in the blood. No statistically significant associations with triglyceride, cholesterol and HDL levels and fat-free mass index were found.

**Table 3:** Metabolic profile at the 24-year follow-up associated with depression in BAMSE

	No depression (*n* = 1570)	Depression (*n* = 301)	Model 1, β [95% CI]^a^	Model 2, β [95% CI]^b^
Bioimpedance at the 24-year follow-up				
Body fat, %	21.83 ± 7.63	23.99 ± 8.11	**1.24 [0.49, 1.99]**	**1.18 [0.42, 1.94]**
Body fat, kg	15.65 ± 7.16	17.25 ± 8.60	**1.22 [0.34, 2.11]**	**1.12 [0.22, 2.01]**
Fat-free mass, kg	55.25 ± 11.05	52.49 ± 9.73	**−1.06 [−1.84, −0.28]**	**−1.10 [−1.89, −0.32]**
Trunk fat, %	20.71 ± 7.00	22.33 ± 7.80	**1.37 [0.49, 2.24]**	**1.31 [0.44, 2.19]**
Fat mass index, kg/m^2^	7.30 ± 2.93	8.20 ± 3.10	**0.50 [0.24, 0.76]**	**0.48 [0.22, 0.74]**
Fat-free mass index, kg/m^2^	17.85 ± 2.19	17.47 ± 2.12	−0.10 [−0.30, 0.11]	−0.12 [−0.32, 0.09]
Blood lipid at the 24-year follow-up	*n* = 1545	*n* = 298		
Triglyceride, mmol/L	1.08 ± 0.57	1.08 ± 0.49	0.03 [−0.04, 0.10]	0.02 [−0.05, 0.09]
Cholesterol, mmol/L	4.20 ± 0.82	4.28 ± 0.82	0.07 [−0.03, 0.16]	0.07 [−0.03, 0.17]
HDL, mmol/L	1.57 ± 0.41	1.60 ± 0.41	−0.03 [−0.07, 0.02]	−0.03 [−0.08, 0.02]
LDL, mmol/L	2.15 ± 0.72	2.20 ± 0.73	0.08 [−0.01, 0.17]	**0.09 [0.00, 0.18]**
LDL/HDL ratio	1.48 ± 0.70	1.49 ± 0.68	**0.09 [0.01, 0.17]**	**0.10 [0.02, 0.18]**

BAMSE, Barn, Allergi, Miljö, Stockholm, Epidemiologi [Children, Allergy, Milieu, Stockholm, Epidemiology]; HDL, high-density lipoprotein; LDL, low-density lipoprotein.

The data were presented as mean ± standard deviation. Statistically significant values are highlighted using bold text.

a. The estimates were adjusted for age, gender and parental education level.

b. The estimates were adjusted for age, gender, parental education level and current smoking status.

### Inflammatory profile

Out of the 92 plasma inflammation-related markers tested (Supplementary Table 4), eight (8.7%) were associated with depression at the nominal significance level (Table 4). After adjusting for potential confounders, such as BMI and smoking, and correcting for multiple testing, four proteins remained significantly associated with depression: fibroblast growth factor 21 (FGF21), FGF19, Interleukin-6 (IL-6) and Interleukin-20 receptor subunit alpha (IL-20RA).

**Table 4:** Systemic inflammation biomarkers at the 24-year follow-up associated with depression in BAMSE

Biomarker^a^	No depression (*n* = 1471)	Depression (*n* = 280)	β [95% CI]^b^	FDR-adjusted *P* value
FGF21	−0.07 ± 0.99	0.19 ± 0.98	0.19 [0.060, 0.312]	**0.021**
IL-6	−0.06 ± 1.00	0.14 ± 0.96	0.17 [0.052, 0.297]	**0.021**
IL-20RA	−0.02 ± 1.01	0.12 ± 0.98	0.16 [0.035, 0.294]	**0.035**
FGF19	−0.03 ± 0.99	0.12 ± 1.07	0.15 [0.023, 0.281]	**0.043**
CX3CL1	−0.03 ± 1.00	0.11 ± 0.98	0.12 [−0.005, 0.253]	0.097
MMP10	−0.02 ± 1.01	0.14 ± 0.94	0.11 [−0.020, 0.236]	0.100
CDCP1	−0.04 ± 0.99	0.11 ± 1.04	0.10 [−0.020, 0.227]	0.100
MCP3	−0.04 ± 1.02	0.11 ± 0.95	0.11 [−0.017, 0.235]	0.100

BAMSE, Barn, Allergi, Miljö, Stockholm, Epidemiologi [Children, Allergy, Milieu, Stockholm, Epidemiology]; FDR, false discovery rate; FGF21, fibroblast growth factor 21; IL-6, interleukin-6; IL-20RA, interleukin-20 receptor subunit alpha; CX3CL1, fractalkine; MMP10, matrix metalloproteinase-10; CDCP1, CUB domain-containing protein 1; MCP3, monocyte chemotactic protein 3.

The data were presented as mean ± standard deviation. Statistically significant values are highlighted using bold text.

a. Data are presented as *z* scores normalised from Normalised Protein eXpression (NPX) values based on inverse normal transformation.

b. The estimates were adjusted for age, gender, body mass index (BMI), parental education level and current smoking status.

### Mediation analyses

We explored the potential mediating effects of inflammatory and metabolic profiles on the association between depression and respiratory symptoms. As top markers, FMI, LDL/HDL ratio and the four proteins with evidence of association with depression in our study were selected as mediators for the analysis. When tested one by one, only fat mass significantly mediated the association (the proportion of mediation was 14.7%, and the 95% Bayesian credible interval = 4.1–37.0%, Supplementary Table 5). Since FGF21 and IL-6 are involved in energy metabolism,^31,32^ we extended the mediation analysis to a hypothesised serial mediation analysis^29^, with inflammatory proteins placed before FMI (Supplementary Fig. 2). We found that FGF21 and FMI together had significant mediating effects on the association between depression and any respiratory symptoms; the total proportion of mediation through all paths with FGF21 and fat max index was 18.3% (Supplementary Table 6). IL-6 together with the FMI also mediated the association between depression and any respiratory symptoms, and the total proportion of mediation was 20.3% (Supplementary Fig. 3 and Table 6).

### Additional analyses

To assess potential unmeasured confounders, we examined a number of childhood factors in relation to depression and found that pneumonia in early childhood was persistently associated with depression (odds ratio = 0.88, 95% CI = 1.13–3.11 for pneumonia during 0–1 years of age, and odds ratio = 1.39, 95% CI = 1.00–1.92 for pneumonia during 0–4 years of age, respectively; see Supplementary Table 7). In another sensitivity analysis, we observed a similar association between depression and respiratory symptoms, with further adjustment for early childhood pneumonia based on model 2 (odds ratio = 1.42, 95% CI = 1.11–1.81, for further adjustment for pneumonia during 0–1 years of age, and odds ratio = 1.39, 95% CI = 1.09–1.78, for further adjustment for pneumonia during 0–4 years of age). Finally, sensitivity analyses of participants who were dispensed with antidepressant medication younger than 18 years and with a wider definition of depression yielded similar results (Supplementary Table 8).

## Discussion

In this study, we found that depression in childhood to young adulthood was associated with higher levels of chronic bronchitis and respiratory symptoms in early adulthood, independently of BMI and smoking status, but was not associated with lung function changes, asthma or allergy. Moreover, depression was associated with metabolic and inflammatory markers in early adulthood, and in mediation analyses, these markers in combination with high body fat index were found to mediate the association between depression and respiratory symptoms. Our results suggested that childhood/adolescent depression may play a role in respiratory health later in life potentially via metabolic and inflammatory pathways.

### Depression associated with respiratory health in early adulthood

Previous studies have found that adulthood depression symptoms are associated with respiratory symptoms.^11,12,14^ Besides, higher future asthma risk has been reported in adult participants with depression, and shared genetic variances between depression and asthma have been suggested.^33^ However, these studies assessed depressive symptoms in adulthood. As childhood/adolescent depression often persists into adulthood,^1,4^ it is plausible that the documented association between adulthood depression and respiratory conditions is partially explained by childhood/adolescent depression. To the best of our knowledge, this is the first study to show that depression during childhood/adolescence is associated with an increased risk of respiratory symptoms in early adulthood. Of note, we observed a stronger association in females than in males. Although females are more likely to develop respiratory symptoms than males,^34^ it is unclear why with the link between depression and respiratory symptoms is particularly stronger in females. Further studies are warranted to understand the underlying reasons. We did not find associations between depression in childhood to early adulthood and asthma, lung function, FeNO, blood eosinophil, neutrophil counts or allergic sensitisation in early adulthood. These results indicate that childhood/adolescent depression is mostly related to later respiratory symptoms, but not asthma features like lung function impairments or airway inflammation. We observed increased chronic bronchitis symptoms and cigarette smoking exposure in depression participants and, more importantly, they are both strong risk factors for chronic obstructive pulmonary disease (COPD) development later in life.^35^ Importantly, depressive symptoms have been reported to be associated with increased risk of chronic lung diseases such as COPD and asthma in middle-aged adults.^36^ Thus, managing respiratory symptoms in this high-risk population might help prevent chronic lung diseases later in life.

### The potential mechanisms of the association

Smoking may be a major contributor to the association with respiratory health outcomes, as patients with depression were more likely to smoke.^13^ However, in the current study, the association between depression and respiratory symptoms remained after adjusting for smoking status. It suggests that smoking cannot explain our findings. In our explorative analysis of potential biological mechanisms underlying the observed relationship, we found that fat mass may partly mediate the association between depression and respiratory symptoms (around 15%). Depression has been associated with increased fat mass and obesity, which may further contribute to shortness of breath and dyspnoea through deteriorated respiratory muscle function and increased oxygen demand required for ventilation.^37^ Together with our results, fat mass is suggested to play a mediating role in the relationship between depression and respiratory symptoms.

Moreover, we found that adding FGF21 and IL-6 to the model, together with fat mass, mediated a greater part of the association (around 18–20%). A limited number of studies have explored the association between depression and FGF21. A previous study^38^ reported increased peripheral FGF21 levels in patients with major depression, while the association between depression and IL-6 has been well documented.^39^ FGF21 is a key hormonal regulator of metabolic function and nutrient preference,^40^ while IL-6 is a pleiotropic protein that targets multiple organs, particularly adipocytes.^32^ That said, both inflammatory proteins play an important role in the regulation of fat mass.^31,32^ Taken together, our results suggest that depression is linked to respiratory symptoms partially through dysregulated metabolic pathways. Further investigation is warranted for potential metabolic changes underlying depression, which may be a shared mechanism for the well documented association between depression, obesity and cardiovascular disease in young adults.^5^

### Strengths and limitations

The major strength of our study is the use of longitudinal data from a large population-based birth cohort with a good follow-up rate from childhood to young adulthood (75% at age 24). Moreover, we used several objective measurements of respiratory health-related clinical characterisations in early adulthood (e.g., spirometry, reversibility test, FeNO and IgE data). In addition, the metabolic and proteomic data allowed us to explore biological mechanisms underlying the noted link.

There are several limitations of this study. First, we identified incident depression through the nationwide Prescribed Drug Register. Patients with depression who did not seek medical help or were not treated with antidepressants were not captured by this approach. However, sensitivity analysis defining depression as either self-reported depression or a dispensation of antidepressants showed similar results. Second, it is possible that some respiratory conditions occurred before depression (i.e., reverse causation). However, our sensitivity analysis restricted to depression before the age of 18 years yielded similar results, which largely alleviates this concern. Third, the serial mediation results should be considered as explorative and interpreted with cautions. Both biomarkers and respiratory outcomes were measured at the 24-year follow-up. The analysis assumes the levels of the biomarkers were largely stable over time or to some extent correlated with the levels in the past. Besides, the current study may have limited statistical power to detect the difference in metabolic profiles. Future studies with larger sample sizes are needed to confirm these findings. Furthermore, while the current study focuses on depression, other mental health issues such as anxiety have also increased in young populations in the last decades. Future studies on mental health conditions other than depression and respiratory health are needed. In addition, the included participants were more likely to be female, had less exposure to maternal smoking during pregnancy, more breastfeeding and less preschool wheezing compared with the participants excluded. However, such selection bias, if any, would have attenuated the association towards null.

## Conclusions

Our findings suggest that depression in childhood to early adulthood is associated with higher levels of respiratory symptoms in early adulthood, independent of unhealthy behaviours. Metabolic and inflammatory dysregulation may underline this link. Health professionals should be aware of the risk of respiratory outcomes in those who exhibit depression in childhood to early adulthood. Future mechanistic research is needed to better understand metabolic and inflammatory pathways underlying this link.

## Supporting information

Wang et al. supplementary material Wang et al. supplementary material

## Data Availability

The data that support the findings of this study are available on reasonable request from the principal investigators of the BAMSE cohort (I.K., A.B. and E.M.). The data are not publicly available due to the privacy and confidentiality of the research participants.

## References

[1] Thapar A, Eyre O, Patel V, Brent D. Depression in young people. Lancet 2022; 400(10352): 617–31. 35940184 10.1016/S0140-6736(22)01012-1

[2] GBD 2019 Diseases and Injuries Collaborators. Global burden of 369 diseases and injuries in 204 countries and territories, 1990–2019: a systematic analysis for the Global Burden of Disease Study 2019. Lancet 2020; 396(10258): 1204–22. 33069326 10.1016/S0140-6736(20)30925-9 PMC7567026

[3] Solmi M, Radua J, Olivola M, Croce E, Soardo L, Salazar de Pablo G, et al. Age at onset of mental disorders worldwide: large-scale meta-analysis of 192 epidemiological studies. Mol Psychiatry 2022; 27(1): 281–95. 34079068 10.1038/s41380-021-01161-7 PMC8960395

[4] Malhi GS, Mann JJ. Depression. Lancet 2018; 392(10161): 2299–312. 30396512 10.1016/S0140-6736(18)31948-2

[5] Goldstein BI, Korczak DJ. Links between child and adolescent psychiatric disorders and cardiovascular risk. Can J Cardiol 2020; 36(9): 1394–405. 32628978 10.1016/j.cjca.2020.06.023

[6] Leone M, Kuja-Halkola R, Leval A, D'Onofrio BM, Larsson H, Lichtenstein P, et al. Association of youth depression with subsequent somatic diseases and premature death. JAMA Psychiatry 2021; 78(3): 302–10. 33295952 10.1001/jamapsychiatry.2020.3786 PMC7726699

[7] European Community Respiratory Health Survey. Variations in the prevalence of respiratory symptoms, self-reported asthma attacks, and use of asthma medication in the European Community Respiratory Health Survey (ECRHS). Eur Respir J 1996; 9(4): 687–95. 8726932 10.1183/09031936.96.09040687

[8] Pleasants RA, Heidari K, Ohar J, Donohue JF, Lugogo NL, Kanotra SM, et al. Respiratory symptoms among US adults: a cross-sectional health survey study. Pulm Ther 2022; 8(3): 255–68. 35794458 10.1007/s41030-022-00194-9 PMC9458821

[9] Cao H, Li S, Baranova A, Zhang F. Shared genetic liability between major depressive disorder and atopic diseases. Front Immunol 2021; 12: 665160. 34566951 10.3389/fimmu.2021.665160 PMC8455950

[10] Zhu Z, Zhu X, Liu CL, Shi H, Shen S, Yang Y, et al. Shared genetics of asthma and mental health disorders: a large-scale genome-wide cross-trait analysis. Eur Respir J 2019; 54(6): 1901507. 31619474 10.1183/13993003.01507-2019

[11] Janson C, Bjornsson E, Hetta J, Boman G. Anxiety and depression in relation to respiratory symptoms and asthma. Am J Respir Crit Care Med 1994; 149(4 Pt 1): 930–4. 8143058 10.1164/ajrccm.149.4.8143058

[12] Neuman A, Gunnbjörnsdottir M, Tunsäter A, Nyström L, Franklin KA, Norrman E, et al. Dyspnea in relation to symptoms of anxiety and depression: a prospective population study. Respir Med 2006; 100(10): 1843–9. 16516455 10.1016/j.rmed.2006.01.016

[13] Ho CSH, Tan ELY, Ho RCM, Chiu MYL. Relationship of anxiety and depression with respiratory symptoms: comparison between depressed and non-depressed smokers in Singapore. Int J Environ Res Public Health 2019; 16(1): 163. 30626156 10.3390/ijerph16010163 PMC6339029

[14] Leander M, Lampa E, Rask-Andersen A, Franklin K, Gislason T, Oudin A, et al. Impact of anxiety and depression on respiratory symptoms. Respir Med 2014; 108(11): 1594–600. 25282543 10.1016/j.rmed.2014.09.007

[15] Beurel E, Toups M, Nemeroff CB. The bidirectional relationship of depression and inflammation: double trouble. Neuron 2020; 107(2): 234–56. 32553197 10.1016/j.neuron.2020.06.002 PMC7381373

[16] Luppino FS, de Wit LM, Bouvy PF, Stijnen T, Cuijpers P, Penninx BWJH, et al. Overweight, obesity, and depression: a systematic review and meta-analysis of longitudinal studies. Arch Gen Psychiatry 2010; 67(3): 220–9. 20194822 10.1001/archgenpsychiatry.2010.2

[17] Garcia-Rio F, Miravitlles M, Soriano JB, Muñoz L, Duran-Tauleria E, Sánchez G, et al. Systemic inflammation in chronic obstructive pulmonary disease: a population-based study. Respiratory Research 2010; 11(1): 63. 20500811 10.1186/1465-9921-11-63 PMC2891677

[18] Baffi CW, Wood L, Winnica D, Strollo PJ, Gladwin MT, Que LG, et al. Metabolic syndrome and the lung. Chest 2016; 149(6): 1525–34. 26836925 10.1016/j.chest.2015.12.034 PMC4944780

[19] Kull I, Melen E, Alm J, Hallberg J, Svartengren M, van Hage M, et al. Breast-feeding in relation to asthma, lung function, and sensitization in young schoolchildren. J Allergy Clin Immunol 2010; 125(5): 1013–9. 20392479 10.1016/j.jaci.2010.01.051

[20] Wang G, Hallberg J, Um Bergstrom P, Janson C, Pershagen G, Gruzieva O, et al. Assessment of chronic bronchitis and risk factors in young adults: results from BAMSE. Eur Respir J 2021; 57(3): 2002120. 33184115 10.1183/13993003.02120-2020 PMC7930470

[21] Wang G, Hallberg J, Faner R, Koefoed HJ, Kebede Merid S, Klevebro S, et al. Plasticity of individual lung function states from childhood to adulthood. Am J Respir Crit Care Med 2023; 207(4): 406–15. 36409973 10.1164/rccm.202203-0444OC PMC9940138

[22] Melen E, Bergstrom A, Kull I, Almqvist C, Andersson N, Asarnoj A, et al. Male sex is strongly associated with IgE-sensitization to airborne but not food allergens: results up to age 24 years from the BAMSE birth cohort. Clin Transl Allergy 2020; 10(1): 15. 32489587 10.1186/s13601-020-00319-w PMC7247167

[23] Wettermark B, Hammar N, Fored CM, Leimanis A, Otterblad Olausson P, Bergman U, et al. The new Swedish Prescribed Drug Register–opportunities for pharmacoepidemiological research and experience from the first six months. Pharmacoepidemiol Drug Saf 2007; 16(7): 726–35. 16897791 10.1002/pds.1294

[24] Wang G, Kull I, Bergstrom A, Hallberg J, Bergstrom PU, Guerra S, et al. Early-life risk factors for reversible and irreversible airflow limitation in young adults: findings from the BAMSE birth cohort. Thorax 2021; 76(5): 503–7. 33184098 10.1136/thoraxjnl-2020-215884 PMC8070638

[25] Quanjer PH, Stanojevic S, Cole TJ, Baur X, Hall GL, Culver BH, et al. Multi-ethnic reference values for spirometry for the 3–95-yr age range: the global lung function 2012 equations. Eur Respir J 2012; 40(6): 1324–43. 22743675 10.1183/09031936.00080312 PMC3786581

[26] Klevebro S, Bjorkander S, Ekstrom S, Merid SK, Gruzieva O, Malarstig A, et al. Inflammation-related plasma protein levels and association with adiposity measurements in young adults. Sci Rep 2021; 11(1): 11391. 34059769 10.1038/s41598-021-90843-x PMC8166979

[27] World Health Organization. The Surveillance of Risk Factors Report Series (SuRF). World Health Organization, 2005.

[28] McCaw ZR, Lane JM, Saxena R, Redline S, Lin X. Operating characteristics of the rank-based inverse normal transformation for quantitative trait analysis in genome-wide association studies. Biometrics 2020; 76(4): 1262–72. 31883270 10.1111/biom.13214 PMC8643141

[29] Hayes AF. Introduction to Mediation, Moderation, and Conditional Process Analysis: A Regression-Based Approach. Guilford Publications, 2022.

[30] Kurz AS. *Recoding Introduction to Mediation, Moderation, and Conditional Process Analysis* version 1.3.0 ed. bookdown.org, 2021 (https://bookdown.org/content/b472c7b3-ede5-40f0-9677-75c3704c7e5c).

[31] Phan P, Saikia BB, Sonnaila S, Agrawal S, Alraawi Z, Kumar TKS, et al. The saga of endocrine FGFs. Cells 2021; 10(9): 2418. 34572066 10.3390/cells10092418 PMC8465397

[32] Qiao Q, Bouwman FG, van Baak MA, Roumans NJT, Vink RG, Mariman ECM. Plasma levels of triglycerides and IL-6 are associated with weight regain and fat mass expansion. J Clin Endocrinol Metabol 2022; 107(7): 1920–9. 10.1210/clinem/dgac198 PMC9202711 35366329

[33] Lehto K, Pedersen NL, Almqvist C, Lu Y, Brew BK. Asthma and affective traits in adults: a genetically informative study. Eur Respir J 2019; 53(5): 1802142. 30956207 10.1183/13993003.02142-2018

[34] Tollefsen E, Langhammer A, Romundstad P, Bjermer L, Johnsen R, Holmen TL. Female gender is associated with higher incidence and more stable respiratory symptoms during adolescence. Respir Med 2007; 101(5): 896–902. 17084607 10.1016/j.rmed.2006.09.022

[35] Global Initiative for Chronic Obstructive Lung Disease (GOLD). *Global Strategy for Diagnosis, Management and Prevention of Chronic Obstructive Lung Disease*. GOLD, 2024 (https://goldcopd.org/2024-gold-report/).

[36] Zheng J, Li J, Pei T, Zhu T, Cheong IH, Li S, et al. Depressive symptoms and chronic lung disease in middle-aged and older Chinese adults: prospective bidirectional association and mediation analysis. J Affect Disord 2022; 297: 283–93. 34688671 10.1016/j.jad.2021.10.023

[37] Zammit C, Liddicoat H, Moonsie I, Makker H. Obesity and respiratory diseases. Int J Gen Med 2010; 3: 335–43. 21116339 10.2147/IJGM.S11926 PMC2990395

[38] Mason BL, Minhajuddin A, Czysz AH, Jha MK, Gadad BS, Mayes TL, et al. Fibroblast growth factor 21 (FGF21) is increased in MDD and interacts with body mass index (BMI) to affect depression trajectory. Transl Psychiatry 2022; 12(1): 16. 35017468 10.1038/s41398-021-01679-y PMC8752780

[39] Kohler CA, Freitas TH, Maes M, de Andrade NQ, Liu CS, Fernandes BS, et al. Peripheral cytokine and chemokine alterations in depression: a meta-analysis of 82 studies. Acta Psychiatr Scand 2017; 135(5): 373–87. 28122130 10.1111/acps.12698

[40] Keipert S, Ost M. Stress-induced FGF21 and GDF15 in obesity and obesity resistance. Trends Endocrinol Metab 2021; 32(11): 904–15. 34526227 10.1016/j.tem.2021.08.008

