# Supplementary material for: Depression in childhood to early adulthood and respiratory health in early adulthood
Source: BJPsych Open. 2024 Nov 11;10(6):e202. doi: 10.1192/bjo.2024.794 (PMC11698180; doi:10.1192/bjo.2024.794)
Supplement: Wang et al. supplementary material [file S2056472424007944sup001.docx]

07 August 2023

Online Data Supplement

**“Depression from childhood to early adulthood and respiratory health in early adulthood”**

Authors:

Gang Wang; Jenny Hallberg; Natalia Hernandez-Pacheco Sandra; Sandra Ekström; Ellen Vercalsteren; Bronwyn K Brew; Catarina Almqvist; Christer Janson; Inger Kull; Anna Bergström; Erik Melén; Donghao Lu

Words: 234; References: 3; Tables: 8; Figure: 3

**ONLINE SUPPLEMENT CONTENTS**

[**METHODS** 3](#_Toc128665016)

[**Definitions of potential confounders and covariates** 3](#_Toc128665017)

[**References** 3](#_Toc128665018)

[**ONLINE SUPPLEMENT TABLES** 4](#_Toc128665019)

[**ONLINE SUPPLEMENT FIGURES** 16](#_Toc128665020)

# **METHODS**

## **Definitions of potential confounders and covariates**

Preterm birth was defined as babies who were delivered before 37 completed weeks of gestation.

Maternal smoking during pregnancy was defined if the mother smoked at least one cigarette per day at any point in time during pregnancy.

Parental education levels were recorded as the highest education of parents at questionnaire 0. The university or higher levels were defined as high level, and the high school or lower levels were defined as low level.

Childhood asthma at ages 0–4 and 8-16 years was defined if at least two of the following three criteria were fulfilled: doctor’s diagnosis of asthma ever; wheezing in the past 12 months; and/or use of asthma medication during the past 12 months (1).

Early life bronchitis, pneumonia, and respiratory syncytial virus were based on parental questionnaire data at each follow-up time point.

Respiratory infections at 24 years of age were assessed based on the question “How many times have you had respiratory infections (a cold with rhinitis, coughing, fever) over the last 12 months?” according to the answers: 1) Never; 2) 1-3 times; 3) 4-6 times; 4) More than 6 times were classified into never, 1-3 times and ≥ 4 times.

BMI z-scores at 4, 8, 12, and 16 years were calculated based on the WHO Growth Reference z-scores (2). Details of the height and weight tests have been described previously (3).

# **References**

1. Wang G, Hallberg J, Um Bergstrom P, Janson C, Pershagen G, Gruzieva O, et al. Assessment of chronic bronchitis and risk factors in young adults: results from BAMSE. Eur Respir J. 2021;57(3).

2. de Onis M. 4.1 The WHO Child Growth Standards. World Rev Nutr Diet. 2015;113:278-94.

3. Ekstrom S, Magnusson J, Kull I, Andersson N, Bottai M, Besharat Pour M, et al. Body Mass Index Development and Asthma Throughout Childhood. Am J Epidemiol. 2017;186(2):255-63.

# **ONLINE SUPPLEMENT TABLES**

**eTable 1. Full names of the 92 proteins included in the Proseek Multiplex Inflammation I panel.**

| **Proteins** | **Full names** |
| --- | --- |
| 4EBP1 | Eukaryotic translation initiation factor 4E-binding protein 1 |
| ADA | Adenosine Deaminase |
| ARTN | Artemin |
| AXIN1 | Axin-1 |
| BetaNGF | Beta-nerve growth factor |
| CASP8 | Caspase-8 |
| CCL3 | C-C motif chemokine 3 |
| CCL4 | C-C motif chemokine 4 |
| CCL11 | C-C motif chemokine 11 |
| CCL19 | C-C motif chemokine 19 |
| CCL20 | C-C motif chemokine 20 |
| CCL23 | C-C motif chemokine 23 |
| CCL25 | C-C motif chemokine 25 |
| CCL28 | C-C motif chemokine 28 |
| CD5 | Cluster of differentiation 5 |
| CD6 | Cluster of differentiation 6 |
| CD8A | Cluster of differentiation 8a |
| CD40 | Cluster of differentiation 40 |
| CD244 | Natural Killer cell receptor 2B4 |
| CDCP1 | CUB domain-containing protein 1 |
| CSF1 | Macrophage colony-stimulating factor 1 |
| CST5 | Cystatin D |
| CX3CL1 | Fractalkine |
| CXCL1 | C-X-C motif chemokine 1 |
| CXCL5 | C-X-C motif chemokine 5 |
| CXCL6 | C-X-C motif chemokine 6 |
| CXCL9 | C-X-C motif chemokine 9 |
| CXCL10 | C-X-C motif chemokine 10 |
| CXCL11 | C-X-C motif chemokine 11 |
| DNER | Delta and Notch-like epidermal or growth factor-related receptor |
| ENRAGE | Protein S100-A12 |
| FGF5 | Fibroblast growth factor 5 |
| FGF19 | Fibroblast growth factor 19 |
| FGF21 | Fibroblast growth factor 21 |
| FGF23 | Fibroblast growth factor 23 |
| Flt3L | Fms-related tyrosine kinase 3 ligand |
| GDNF | Glial cell line-derived neurotrophic factor |
| HGF | Hepatocyte growth factor |
| IFNgamma | Interferon-gamma |
| IL1 alpha | Interleukin-1 alpha |
| IL2 | Interleukin-2 |
| IL2RB | Interleukin-2 receptor subunit beta |
| IL4 | Interleukin-4 |
| IL5 | nterleukin-5 |
| IL6 | Interleukin-6 |
| IL7 | Interleukin-7 |
| IL8 | Interleukin-8 |
| IL10 | Interleukin-10 |
| IL10RA | Interleukin-10 receptor subunit alpha |
| IL10RB | Interleukin-10 receptor beta |
| IL12B | Interleukin-12 subunit beta |
| IL13 | Interleukin-13 |
| IL15RA | Interleukin-15 receptor alpha |
| IL17A | Interleukin-17A |
| IL17C | Interleukin-17C |
| IL18 | Interleukin-18 |
| IL18R1 | Interleukin-18 receptor 1 |
| IL20 | nterleukin-20 |
| IL20RA | Interleukin-20 receptor subunit alpha |
| IL22 RA1 | Interleukin-22 receptor subunit alpha-1 |
| IL24 | Interleukin-24 |
| IL33 | Interleukin-33 |
| LAPTGFbeta1 | Latency-associated peptide transforming or growth factor beta-1 |
| LIF | Leukemia inhibitory factor |
| LIFR | Leukemia inhibitory factor receptor |
| MCP1 | Monocyte chemotactic protein 1 |
| MCP2 | Monocyte chemotactic protein 2 |
| MCP3 | Monocyte chemotactic protein 3 |
| MCP4 | Monocyte chemotactic protein 4 |
| MMP1 | Matrix metalloproteinase-1 |
| MMP10 | Matrix metalloproteinase-10 |
| NRTN | Neurturin |
| NT3 | Neurotrophin-3 |
| OPG | Osteoprotegerin |
| OSM | Oncostatin-M |
| PDL1 | Programmed cell death ligand 1 |
| SCF | Stem Cell Factor |
| SIRT2 | SIR2-like protein 2 |
| SLAMF1 | Signaling lymphocytic activation molecule |
| ST1A1 | Sulfotransferase 1A1 |
| STAMBP | STAM-binding protein |
| TGFalpha | Transforming Growth Factor alpha |
| TNF | Tumor necrosis factor |
| TNFB | Tumor necrosis factor beta |
| TNFRSF9 | Tumor necrosis factor receptor superfamily member 9 |
| TNFSF14 | Tumor necrosis factor ligand superfamily member 14 |
| TRAIL | TNF-related apoptosis-inducing ligand |
| TRANCE | TNF-related activation-induced cytokine |
| TSLP | Thymic stromal lymphopoietin |
| TWEAK | TNF-related weak inducer of apoptosis |
| uPA | Urokinase-type plasminogen activator |
| VEGFA | Vascular endothelial growth factor A |

**eTable 2. Characteristics of participants been included in or excluded from the analysis.**

|  | Included (N=2994) | Excluded (N=1095) | P value |
| --- | --- | --- | --- |
| Sex |  |  |  |
| Female | 1586 (53.0%) | 438 (40.0%) | <0.001 |
| Male | 1408 (47.0%) | 657 (60.0%) |  |
| Highest parental education |  |  |  |
| University | 1666 (55.7%) | 495 (45.3%) | <0.001 |
| Primary school/High school | 1324 (44.3%) | 597 (54.7%) |  |
| BMI z-scores |  |  |  |
| 4 years | 0.61 ± 0.89 | 0.64 ± 0.91 | 0.49 |
| 8 years | 0.58 ± 0.97 | 0.59 ± 1.02 | 0.89 |
| 12 years | -0.07 ± 1.08 | -0.04 ± 1.12 | 0.62 |
| 16 years | 0.12 ± 0.96 | 0.11 ± 1.03 | 0.95 |
| Obesity |  |  |  |
| 4 years | 285 (11.2%) | 93 (12.8%) | 0.21 |
| 8 years | 301 (14.2%) | 76 (15.2%) | 0.57 |
| 12 years | 143 (6.0%) | 44 (7.9%) | 0.096 |
| 16 years | 154 (6.0%) | 39 (7.2%) | 0.28 |
| Asthma |  |  |  |
| Pre-school age (0-4y) | 654 (22.1%) | 256 (24.7%) | 0.085 |
| School age (8-16y) | 500 (18.1%) | 133 (16.5%) | 0.31 |
| Wheezing > 3 times |  |  |  |
| Pre-school age (0-4y) | 424 (14.3%) | 182 (17.6%) | 0.013 |
| School age (8-16y) | 252 (9.1%) | 78 (9.4%) | 0.76 |
| Early life events |  |  |  |
| Premature birth | 166 (5.5%) | 61 (5.6%) | 0.97 |
| Maternal smoking during pregnancy | 355 (11.9%) | 172 (15.7%) | 0.0012 |
| Birth weight, Kg | 3.53 ± 0.56 | 3.53 ± 0.56 | 0.94 |
| Exclusive breast feeding for more than 4 months | 2336 (80.4%) | 780 (77.1%) | 0.026 |
| Bronchitis during 0-1 years | 221 (7.6%) | 80 (7.9%) | 0.78 |
| Pneumonia during 0-1 years | 99 (3.4%) | 43 (4.2%) | 0.22 |
| RSV infections during 0-1 years | 133 (4.6%) | 32 (3.2%) | 0.053 |
| Pneumonia during 0-4 years | 334 (11.3%) | 113 (10.9%) | 0.73 |

**eTable 3. Association between depression and respiratory symptoms at age 24,** **stratified by sex, BMI, and asthma.**

| * | No depression | Depression |  |
| --- | --- | --- | --- |
|  | respiratory symptoms, N (%) | respiratory symptoms, N (%) | OR [95% CI] |
| By sex |  |  |  |
| Female | 251 (23.2%) | 88 (35.6%) | 1.73 [1.28, 2.35] |
| Male | 219 (20.5%) | 35 (22.4%) | 1.01 [0.67, 1.53] |
| P for interaction | 0.046 |  |  |
|  |  |  |  |
| By BMI |  |  |  |
| Normal/underweight | 329 (20.1%) | 82 (28.6%) | 1.48 [1.11, 1.98] |
| Overweight/obesity | 141 (28.4%) | 39 (34.8%) | 1.24 [0.80, 1.93] |
| P for interaction | 0.61 |  |  |
|  |  |  |  |
| By asthma |  |  |  |
| No | 192 (10.4%) | 54 (16.2%) | 1.49 [1.06, 2.08] |
| Yes | 276 (94.2%) | 69 (98.6%) | 3.25 [0.41, 25.51] |
| P for interaction | 0.39 |  |  |

* Results after adjustment for age, sex, parental education level, BMI, and current smoking status.

**eTable 4. Levels of 84 plasma inflammatory markers across depression state.**

| Names^*^ | Depression (n=280) | Non-depression (n=1471) | P value |
| --- | --- | --- | --- |
| ADA | 0.00 ± 1.00 | -0.03 ± 0.97 | 0.652 |
| ARTN | 0.01 ± 1.01 | 0.02 ± 1.00 | 0.852 |
| AXIN1 | -0.00 ± 1.00 | -0.01 ± 1.00 | 0.986 |
| BetaNGF | -0.00 ± 1.01 | -0.02 ± 0.94 | 0.754 |
| CASP8 | 0.00 ± 1.01 | -0.07 ± 0.97 | 0.265 |
| CCL3 | -0.01 ± 0.99 | -0.00 ± 0.99 | 0.865 |
| CCL4 | -0.01 ± 1.00 | -0.02 ± 1.03 | 0.826 |
| CCL11 | -0.02 ± 1.01 | 0.09 ± 0.96 | 0.088 |
| CCL19 | -0.03 ± 0.98 | 0.09 ± 1.04 | 0.083 |
| CCL20 | -0.02 ± 1.00 | -0.01 ± 1.05 | 0.841 |
| CCL23 | 0.01 ± 1.00 | -0.03 ± 0.94 | 0.457 |
| CCL25 | -0.03 ± 1.01 | 0.05 ± 1.00 | 0.206 |
| CCL28 | -0.02 ± 0.99 | 0.03 ± 1.01 | 0.479 |
| CD5 | -0.01 ± 1.00 | -0.01 ± 0.95 | 0.970 |
| CD6 | -0.01 ± 1.01 | -0.02 ± 0.92 | 0.940 |
| CD8A | 0.01 ± 1.00 | 0.06 ± 0.96 | 0.467 |
| CD40 | -0.01 ± 0.99 | -0.05 ± 1.01 | 0.518 |
| CD244 | -0.01 ± 0.99 | -0.06 ± 0.98 | 0.406 |
| CSF1 | 0.02 ± 1.01 | -0.07 ± 0.93 | 0.173 |
| CST5 | -0.01 ± 0.99 | -0.06 ± 0.97 | 0.433 |
| CXCL1 | 0.00 ± 1.00 | 0.00 ± 1.03 | 0.988 |
| CXCL5 | -0.01 ± 1.00 | 0.03 ± 1.00 | 0.559 |
| CXCL6 | 0.01 ± 0.99 | -0.04 ± 1.00 | 0.516 |
| CXCL9 | 0.01 ± 1.01 | 0.02 ± 0.98 | 0.862 |
| CXCL10 | 0.01 ± 1.00 | 0.00 ± 1.03 | 0.881 |
| CXCL11 | -0.02 ± 1.01 | 0.06 ± 0.95 | 0.225 |
| DNER | 0.00 ± 1.00 | 0.04 ± 1.01 | 0.519 |
| EBP1 | -0.02 ± 1.00 | -0.00 ± 0.97 | 0.784 |
| ENRAGE | -0.00 ± 1.00 | 0.01 ± 1.00 | 0.872 |
| FGF5 | -0.01 ± 1.00 | 0.02 ± 0.99 | 0.657 |
| FGF23 | -0.04 ± 0.98 | 0.09 ± 1.03 | 0.054 |
| Flt3L | -0.00 ± 0.99 | 0.08 ± 1.02 | 0.175 |
| GDNF | -0.04 ± 1.01 | 0.09 ± 0.96 | 0.059 |
| HGF | -0.05 ± 1.02 | 0.01 ± 0.92 | 0.367 |
| IFNgamma | 0.02 ± 1.01 | -0.05 ± 1.02 | 0.242 |
| IL1alpha | -0.01 ± 1.00 | -0.01 ± 0.99 | 0.928 |
| IL2 | -0.02 ± 1.00 | 0.04 ± 0.98 | 0.381 |
| IL2RB | 0.03 ± 1.00 | 0.00 ± 0.97 | 0.655 |
| IL4 | 0.00 ± 1.00 | -0.07 ± 1.04 | 0.241 |
| IL5 | -0.00 ± 1.01 | 0.01 ± 1.04 | 0.878 |
| IL7 | -0.00 ± 1.00 | 0.01 ± 1.01 | 0.867 |
| IL8 | -0.02 ± 1.00 | -0.01 ± 0.98 | 0.959 |
| IL10 | -0.02 ± 0.99 | 0.07 ± 1.01 | 0.158 |
| IL10RA | 0.00 ± 0.99 | -0.09 ± 1.04 | 0.189 |
| IL10RB | -0.02 ± 1.00 | 0.06 ± 1.00 | 0.211 |
| IL12B | 0.01 ± 0.99 | -0.01 ± 1.04 | 0.846 |
| IL13 | 0.02 ± 1.00 | -0.08 ± 0.94 | 0.130 |
| IL15RA | 0.00 ± 1.01 | -0.05 ± 0.97 | 0.459 |
| IL17A | 0.00 ± 1.00 | -0.04 ± 1.03 | 0.565 |
| IL17C | -0.01 ± 1.02 | 0.04 ± 0.95 | 0.418 |
| IL18 | -0.02 ± 1.00 | 0.06 ± 0.96 | 0.218 |
| IL18R1 | -0.02 ± 1.00 | 0.06 ± 0.98 | 0.226 |
| IL20 | -0.01 ± 1.00 | -0.01 ± 0.97 | 0.988 |
| IL22RA1 | -0.00 ± 1.00 | 0.02 ± 0.95 | 0.700 |
| IL24 | 0.02 ± 1.00 | -0.03 ± 1.01 | 0.412 |
| IL33 | 0.00 ± 1.00 | -0.07 ± 0.99 | 0.239 |
| LAPTGFbeta1 | -0.01 ± 1.01 | -0.05 ± 0.96 | 0.508 |
| LIF | -0.01 ± 1.01 | 0.03 ± 0.93 | 0.551 |
| LIFR | -0.01 ± 1.01 | 0.03 ± 0.97 | 0.549 |
| MCP1 | -0.04 ± 0.99 | 0.07 ± 1.03 | 0.078 |
| MCP2 | -0.01 ± 1.02 | -0.05 ± 0.92 | 0.528 |
| MCP4 | -0.03 ± 1.00 | 0.03 ± 0.99 | 0.366 |
| MMP1 | -0.01 ± 1.00 | -0.02 ± 0.97 | 0.927 |
| NRTN | 0.02 ± 0.99 | -0.06 ± 0.99 | 0.178 |
| NT3 | 0.02 ± 0.99 | -0.09 ± 1.06 | 0.094 |
| OPG | 0.01 ± 1.01 | 0.02 ± 0.98 | 0.930 |
| OSM | -0.01 ± 1.00 | -0.01 ± 0.92 | 0.989 |
| PDL1 | 0.02 ± 1.00 | -0.08 ± 0.99 | 0.138 |
| SCF | -0.01 ± 0.99 | 0.02 ± 0.93 | 0.590 |
| SIRT2 | -0.01 ± 1.00 | -0.00 ± 1.00 | 0.922 |
| SLAMF1 | -0.00 ± 1.02 | -0.01 ± 0.94 | 0.902 |
| ST1A1 | 0.00 ± 1.00 | 0.01 ± 0.97 | 0.946 |
| STAMBP | -0.01 ± 1.00 | -0.02 ± 1.00 | 0.819 |
| TGFalpha | -0.04 ± 1.01 | 0.08 ± 0.93 | 0.069 |
| TNF | -0.01 ± 1.00 | -0.00 ± 1.01 | 0.883 |
| TNFB | 0.01 ± 1.00 | 0.00 ± 0.97 | 0.950 |
| TNFRSF9 | -0.00 ± 0.98 | -0.00 ± 1.01 | 0.959 |
| TNFSF14 | -0.01 ± 1.02 | -0.04 ± 0.93 | 0.676 |
| TRAIL | -0.02 ± 1.02 | 0.09 ± 0.95 | 0.100 |
| TRANCE | -0.02 ± 1.00 | -0.01 ± 0.90 | 0.864 |
| TSLP | 0.01 ± 1.01 | -0.07 ± 0.98 | 0.216 |
| TWEAK | -0.03 ± 1.02 | -0.01 ± 0.94 | 0.801 |
| uPA | -0.03 ± 1.01 | 0.08 ± 0.97 | 0.091 |
| VEGFA | -0.01 ± 0.99 | 0.00 ± 0.94 | 0.808 |

^*^: Data are presented as Normalized Protein eXpression (NPX) values, which are Olink Proteomics’ arbitrary units on a log2 scale.

**eTable 5. Potential mediation effect of metabolic and inflammatory profiles on the association between depression and respiratory symptoms.**

| Mediator^a^ | Direct effect | | Indirect effect | | Total effect | | Proportion of mediation | |
| --- | --- | --- | --- | --- | --- | --- | --- | --- |
|  | Estimate | 95% CI* | Estimate | 95% CI* | Estimate | 95% CI* | Mean and median (%) | 95% CI* |
| Fat mass index | 0.249 | [-0.023, 0.528] | 0.049 | [0.017, 0.089] | 0.387 | [0.147, 0.618] | 14.7, 12.3 | [4.1, 37.0] |
| LDL/HDL ratio | 0.277 | [-0.010, 0.556] | 0.014 | [-0.002, 0.039] | 0.387 | [0.147, 0.618] | - | - |
| FGF21 | 0.314 | [0.022, 0.598] | 0.007 | [-0.016, 0.034] | 0.387 | [0.147, 0.618] | - | - |
| FGF19 | 0.325 | [0.042, 0.616] | -0.005 | [-0.026, 0.011] | 0.387 | [0.147, 0.618] | - | - |
| IL-6 | 0.312 | [0.023, 0.607] | 0.014 | [-0.009, 0.044] | 0.387 | [0.147, 0.618] | - | - |
| IL-20RA | 0.317 | [0.030, 0.599] | 0.008 | [-0.012, 0.032] | 0.387 | [0.147, 0.618] | - | - |

LDL: low-density lipoprotein; HDL: high-density lipoprotein; FGF21: fibroblast growth factor 21; FGF19: fibroblast growth factor 19; IL-6: Interleukin-6; IL-20RA: Interleukin-20 receptor subunit alpha.

*: 95% Bayesian credible interval.

^a^: the models were adjusted for age, sex, parental education level, and smoking status.

**eTable 6. Total, direct, and indirect effects of depression on respiratory symptoms (n = 1742)**

|  |  |  | Effect | | The ratio of indirect to total effect* | |
| --- | --- | --- | --- | --- | --- | --- |
| By FEG21 and fat mass index |  |  | Mean and median | 95% CI | Mean and median | 95% CI |
| Total effect |  | c | 0.386, 0.389 | [0.142, 0.623] |  | - |
| Direct effect |  | c’ | 0.274, 0.273 | [ -0.020, 0.562] |  | - |
| Indirect effect |  |  |  |  |  |  |
|  | Via FGF 21 only | a1*b1 | -0.004, -0.003 | [-0.029, 0.021] |  | - |
|  | Via fat mass index only | a2*b2 | 0.046, 0.044 | [0.015, 0.087] | 15.2%, 11.6% | [3.4%, 36.0%] |
|  | Via FGF 21 and fat mass index | a1*d21*b2 | 0.010, 0.009 | [0.003, 0.020] | 3.1%, 2.4% | [0.6%, 8.4%] |
|  |  |  |  |  |  |  |
| By IL-6 and fat mass index |  |  |  |  |  |  |
| Total effect |  | c | 0.385, 0.385 | [0.140, 0.619] |  | - |
| Direct effect |  | c’ | 0.273, 0.273 | [-0.010, 0.569] |  | - |
| Indirect effect |  |  |  |  |  |  |
|  | Via IL-6 only | a1*b1 | -0.005, -0.005 | [-0.033, 0.022] |  | - |
|  | Via fat mass index only | a2*b2 | 0.039, 0.037 | [0.009 , 0.078] | 13.9%, 9.8% | [2.2%, 33.5%] |
|  | Via IL-6 and fat mass index | a1*d21*b2 | 0.018, 0.017 | [0.005 , 0.034] | 6.4%, 4.4% | [1.3%, 14.8%] |

*: based on the absolute values.

**eTable 7. Potential risk factors during childhood associated with depression in BAMSE.**

|  | No depression (n=2150) | Depression (n=403) | Model 1, OR [95% CI]^a^ | Model 2, OR [95% CI]^b^ |
| --- | --- | --- | --- | --- |
| BMI z-scores |  |  |  |  |
| 4 years | 0.60 ± 0.88 | 0.69 ± 0.93 | **1.15 [1.01, 1.31]** | 1.09 [0.94, 1.26] |
| 8 years | 0.56 ± 0.97 | 0.64 ± 0.94 | 1.10 [0.96, 1.26] | 1.02 [0.86, 1.20] |
| 12 years | -0.08 ± 1.08 | 0.00 ± 1.08 | 1.10 [0.98, 1.24] | 1.02 [0.88, 1.18] |
| 16 years | 0.11 ± 0.94 | 0.12 ± 1.05 | 1.03 [0.91, 1.16] | 0.92 [0.77, 1.09] |
| Obesity |  |  |  |  |
| 4 years | 198 (10.7%) | 49 (15.0%) | **1.53 [1.09, 2.14]** | 1.40 [0.97, 2.01] |
| 8 years | 211 (13.4%) | 39 (15.4%) | 1.21 [0.84, 1.76] | 1.04 [0.68, 1.59] |
| 12 years | 98 (5.7%) | 19 (6.1%) | 1.17 [0.70, 1.96] | 0.84 [0.46, 1.52] |
| 16 years | 101 (5.4%) | 21 (6.4%) | 1.27 [0.78, 2.07] | 1.06 [0.61, 1.86] |
| Asthma |  |  |  |  |
| Pre-school age (0-4y) | 467 (22.0%) | 91 (22.7%) | 1.09 [0.84, 1.41] | 1.06 [0.82, 1.38] |
| School age (8-16y) | 366 (18.3%) | 65 (17.7%) | 0.98 [0.73, 1.31] | 0.95 [0.71, 1.28] |
| Wheezing > 3 times |  |  |  |  |
| Pre-school age (0-4y) | 314 (14.8%) | 50 (12.5%) | 0.86 [0.62, 1.18] | 0.83 [0.60, 1.15] |
| School age (8-16y) | 187 (9.3%) | 34 (9.2%) | 1.01 [0.68, 1.48] | 0.95 [0.64, 1.40] |
| Early life events |  |  |  |  |
| Premature birth | 124 (5.8%) | 14 (3.5%) | 0.59 [0.34, 1.04] | 0.62 [0.35, 1.10] |
| Maternal smoking during pregnancy | 247 (11.5%) | 51 (12.7%) | 1.11 [0.80, 1.55] | 0.97 [0.69, 1.36] |
| Birth weight, Kg | 3.53 ± 0.56 | 3.56 ± 0.56 | 1.18 [0.97, 1.43] | 1.14 [0.94, 1.40] |
| Exclusive breast feeding for more than 4 months | 1666 (79.7%) | 324 (81.8%) | 1.16 [0.88, 1.53] | 1.21 [0.91, 1.60] |
| Bronchitis during 0-1 years | 160 (7.7%) | 34 (8.6%) | 1.14 [0.78, 1.69] | 1.16 [0.78, 1.71] |
| Pneumonia during 0-1 years | 65 (3.1%) | 22 (5.6%) | **1.88 [1.14, 3.10]** | **1.88 [1.13, 3.11]** |
| Respiratory syncytial virus infections during 0-1 years | 99 (4.7%) | 17 (4.3%) | 0.82 [0.48, 1.41] | 0.81 [0.47, 1.39] |
| Pneumonia during 0-4 years | 220 (10.4%) | 54 (13.5%) | **1.38 [1.00, 1.90]** | **1.39 [1.00, 1.92]** |

*: The estimates were adjusted for age, sex, and parental education level.

^a^: The estimates were adjusted for age, sex, and parental education level.

^b^: The estimates were adjusted for age, sex, parental education level, BMI, and current smoking status.

**eTable 8. Sensitivity analysis.**

| **Sensitivity analysis 1^a^** | No depression (n=2150) | Depression (n=126) | Model 1, OR [95% CI]^e^ | Model 2, OR [95% CI]^f^ |
| --- | --- | --- | --- | --- |
| Respiratory symptoms |  |  |  |  |
| Any symptom | 470 (21.9%) | 41 (32.5%) | **1.65 [ 1.12, 2.43]** | **1.53 [ 1.03, 2.27]** |
| Wheezing > 3 times | 232 (10.8%) | 27 (21.4%) | **2.11 [ 1.35, 3.32]** | **2.09 [ 1.33, 3.29]** |
| Asthma | 293 (13.6%) | 20 (15.9%) | 1.14 [ 0.69, 1.87] | 1.11 [ 0.67, 1.84] |
| Respiratory infections |  |  |  |  |
| 1-3 times | 1341 (65.9%) | 84 (68.3%) | 1.09 [0.62, 1.92] | 1.05 [0.59, 1.86] |
| More than 4 times | 427 (21.0%) | 24 (19.5%) | 0.91 [0.47, 1.79] | 0.90 [0.46, 1.78] |
| Chronic bronchitis symptoms |  |  |  |  |
| Any chronic bronchitis symptoms | 322 (15.9%) | 25 (20.5%) | 1.36 [ 0.86, 2.15] | 1.30 [ 0.82, 2.07] |
| Only Cough | 57 (2.8%) | 2 (1.6%) | 0.59 [0.14, 2.46] | 0.55 [0.13, 2.32] |
| Only Mucus | 174 (8.6%) | 17 (13.9%) | **1.75 [1.02, 3.02]** | **1.74 [1.01, 3.00]** |
| Chronic bronchitis | 91 (4.5%) | 6 (4.9%) | 1.11 [0.47, 2.61] | 1.01 [0.42, 2.41] |
|  |  |  |  |  |
| **Sensitivity analysis 2^b^** | No depression (n=2150) | Depression (n= 844) | Model 1, OR [95% CI]^a^ | Model 2, OR [95% CI]^b^ |
| Respiratory symptoms |  |  |  |  |
| Any symptom | 470 (21.9%) | 249 (29.5%) | **1.45 [ 1.21, 1.74]** | **1.38 [ 1.15, 1.66]** |
| Wheezing > 3 times | 232 (10.8%) | 144 (17.1%) | **1.65 [1.32, 2.07]** | **1.62 [1.29, 2.04]** |
| Asthma | 293 (13.6%) | 124 (14.7%) | 1.06 [0.84, 1.33] | 1.03 [0.82, 1.30] |
| Respiratory infections |  |  |  |  |
| 1-3 times | 1341 (65.9%) | 494 (61.1%) | 0.92 [0.72, 1.18] | 0.93 [0.72, 1.20] |
| More than 4 times | 427 (21.0%) | 208 (25.7%) | 1.18 [0.89, 1.57] | 1.18 [0.88, 1.57] |
| Chronic bronchitis symptoms |  |  |  |  |
| Any chronic bronchitis symptoms | 322 (15.9%) | 191 (23.8%) | **1.67 [ 1.36, 2.04]** | **1.56 [ 1.27, 1.92]** |
| Only Cough | 57 (2.8%) | 34 (4.2%) | **1.65 [1.06, 2.56]** | 1.53 [0.98, 2.39] |
| Only Mucus | 174 (8.6%) | 95 (11.8%) | **1.55 [1.19, 2.03]** | **1.49 [1.14, 1.96]** |
| Chronic bronchitis | 91 (4.5%) | 62 (7.7%) | **1.91 [1.36, 2.68]** | **1.71 [1.21, 2.42]** |
|  |  |  |  |  |
| **Sensitivity analysis 3^c^** | No depression (n=2150) | Depression (n=691) | Model 1, OR [95% CI]^a^ | Model 2, OR [95% CI]^b^ |
| Respiratory symptoms |  |  |  |  |
| Any symptom | 470 (21.9%) | 213 (30.8%) | **1.55 [ 1.28, 1.88]** | **1.47 [ 1.21, 1.79]** |
| Wheezing > 3 times | 232 (10.8%) | 124 (18.0%) | **1.76 [ 1.39, 2.23]** | **1.72 [ 1.35, 2.19]** |
| Asthma | 293 (13.6%) | 107 (15.5%) | 1.13 [ 0.89, 1.44] | 1.09 [ 0.85, 1.40] |
| Respiratory infections |  |  |  |  |
| 1-3 times | 1341 (65.9%) | 400 (60.3%) | 0.91 [0.70, 1.19] | 0.92 [0.70, 1.20] |
| More than 4 times | 427 (21.0%) | 177 (26.7%) | 1.24 [0.92, 1.68] | 1.23 [0.91, 1.68] |
| Chronic bronchitis symptoms |  |  |  |  |
| Any chronic bronchitis symptoms | 322 (15.9%) | 165 (25.1%) | **1.78 [1.44, 2.21]** | **1.67 [1.34, 2.07]** |
| Only Cough | 57 (2.8%) | 26 (4.0%) | 1.57 [0.97, 2.53] | 1.45 [0.89, 2.35] |
| Only Mucus | 174 (8.6%) | 84 (12.8%) | **1.69 [1.28, 2.24]** | **1.64 [1.23, 2.17]** |
| Chronic bronchitis | 91 (4.5%) | 55 (8.4%) | **2.09 [1.47, 2.97]** | **1.86 [1.30, 2.67]** |
|  |  |  |  |  |
| **Sensitivity analysis 4^d^** | No depression (n=2150) | Depression (n=556) | Model 1, OR [95% CI]^a^ | Model 2, OR [95% CI]^b^ |
| Respiratory symptoms |  |  |  |  |
| Any symptom | 470 (21.9%) | 159 (28.6%) | **1.39 [ 1.13, 1.72]** | **1.30 [ 1.04, 1.61]** |
| Wheezing > 3 times | 232 (10.8%) | 97 (17.4%) | **1.70 [ 1.31, 2.20]** | **1.66 [ 1.28, 2.16]** |
| Asthma | 293 (13.6%) | 87 (15.6%) | 1.15 [ 0.88, 1.49] | 1.09 [ 0.84, 1.43] |
| Respiratory infections |  |  |  |  |
| 1-3 times | 1341 (65.9%) | 340 (63.8%) | 1.02 [0.76, 1.37] | 1.04 [0.77, 1.41] |
| More than 4 times | 427 (21.0%) | 127 (23.8%) | 1.16 [0.83, 1.63] | 1.17 [0.83, 1.65] |
| Chronic bronchitis symptoms |  |  |  |  |
| Any chronic bronchitis symptoms | 322 (15.9%) | 120 (22.6%) | **1.56 [1.23, 1.98]** | **1.44 [1.13, 1.84]** |
| Only Cough | 57 (2.8%) | 22 (4.1%) | 1.58 [0.95, 2.63] | 1.46 [0.87, 2.45] |
| Only Mucus | 174 (8.6%) | 63 (11.8%) | **1.53 [1.12, 2.09]** | **1.46 [1.07, 2.00]** |
| Chronic bronchitis | 91 (4.5%) | 35 (6.6%) | **1.62 [1.07, 2.43]** | **1.40 [0.92, 2.13]** |

OR: odds ratio; CI: confidence interval.

a: Only participants with anti-depressant medication younger than 18 years were included.

b: The depression was defined as a record of antidepressant prescription or self-reported depression.

c: The depression was defined as a record of antidepressant prescription and self-reported depression or self-reported depression.

d: The depression was defined as a record of antidepressant prescription and self-reported depression or a record of antidepressant prescription.

^e^: The estimates were adjusted for age, sex, and parental education level.

^f^: The estimates were adjusted for age, sex, parental education level, BMI, and current smoking status.

# **ONLINE SUPPLEMENT FIGURES**


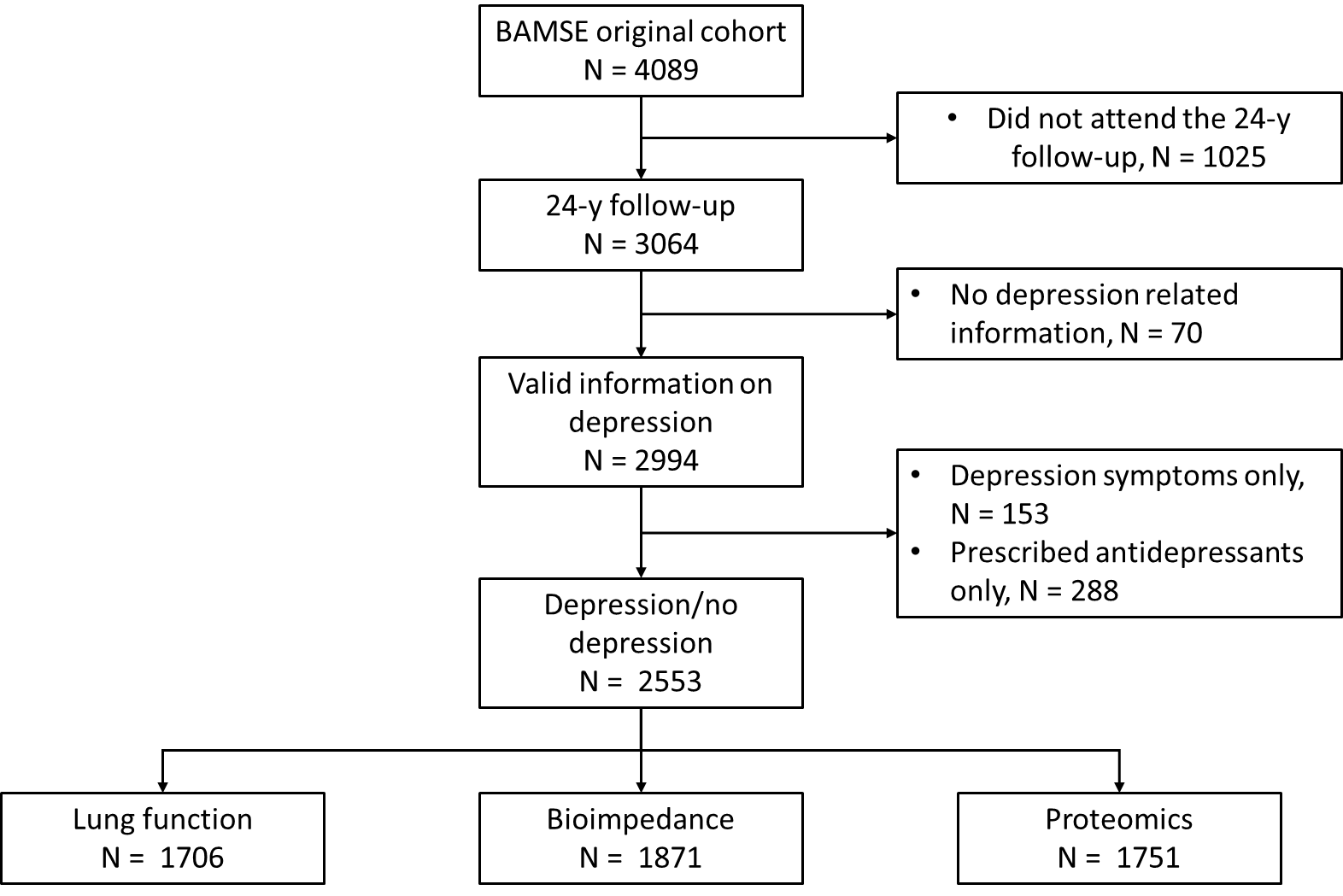


**eFigure 1. Study design.**


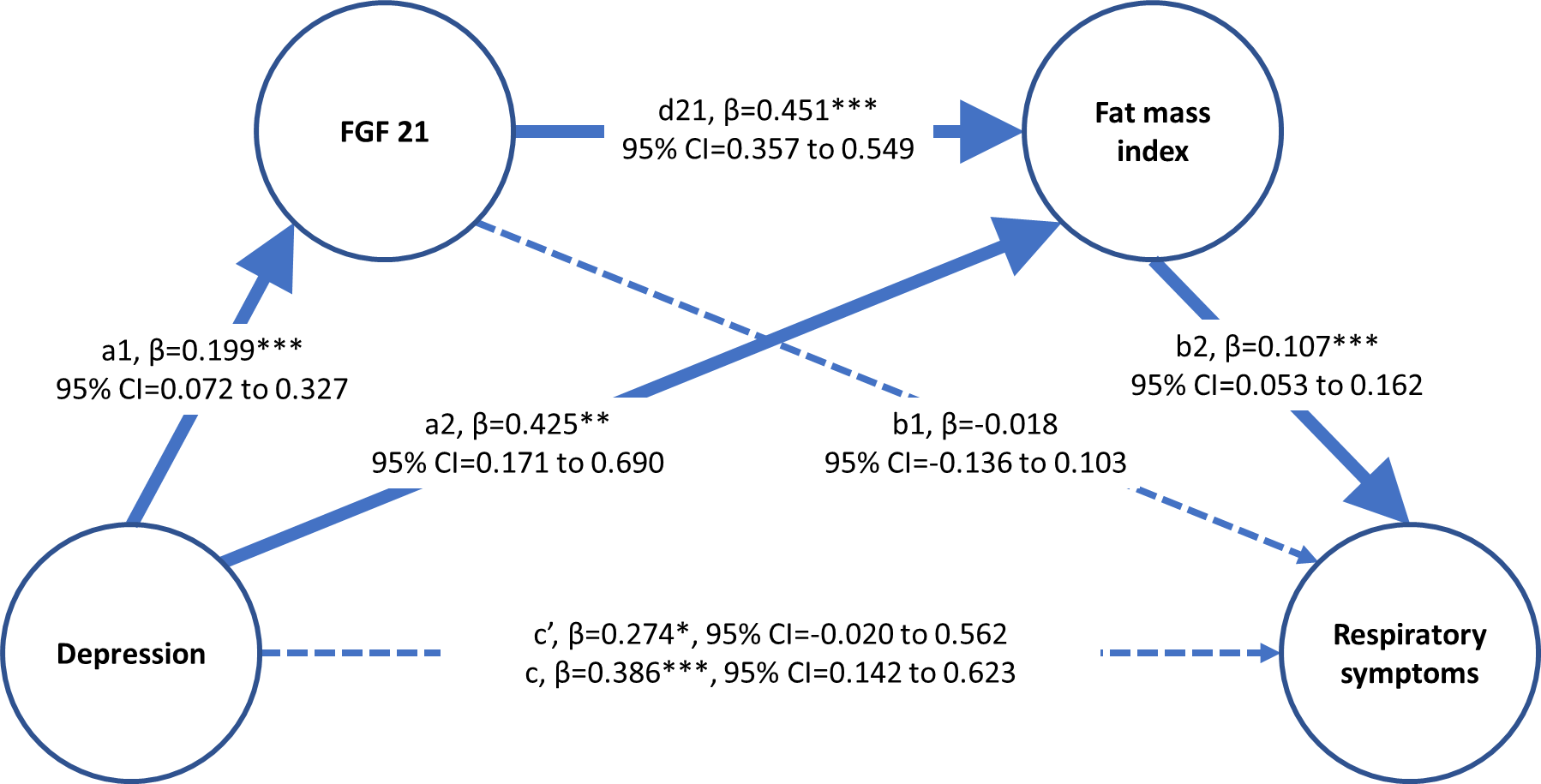


**eFigure 2. Serial mediation model hypothesis linking depression and respiratory symptoms (n=1742).** Effects were estimated using Bayesian based methods and presented as posterior mean with percentile-based 95% Bayesian credible interval of β (95% CI). The indirect effect (such as a1*b1, a2*b2, and a1*d21*b2) and the ratio of indirect to total effect is presented in eTable 6.

c: total effect; c’: total direct effect; *: the proportion of Bayesian credible interval less than 0 (Bayesian p value) <0.05; **: p<0.01; ***: p<0.001.


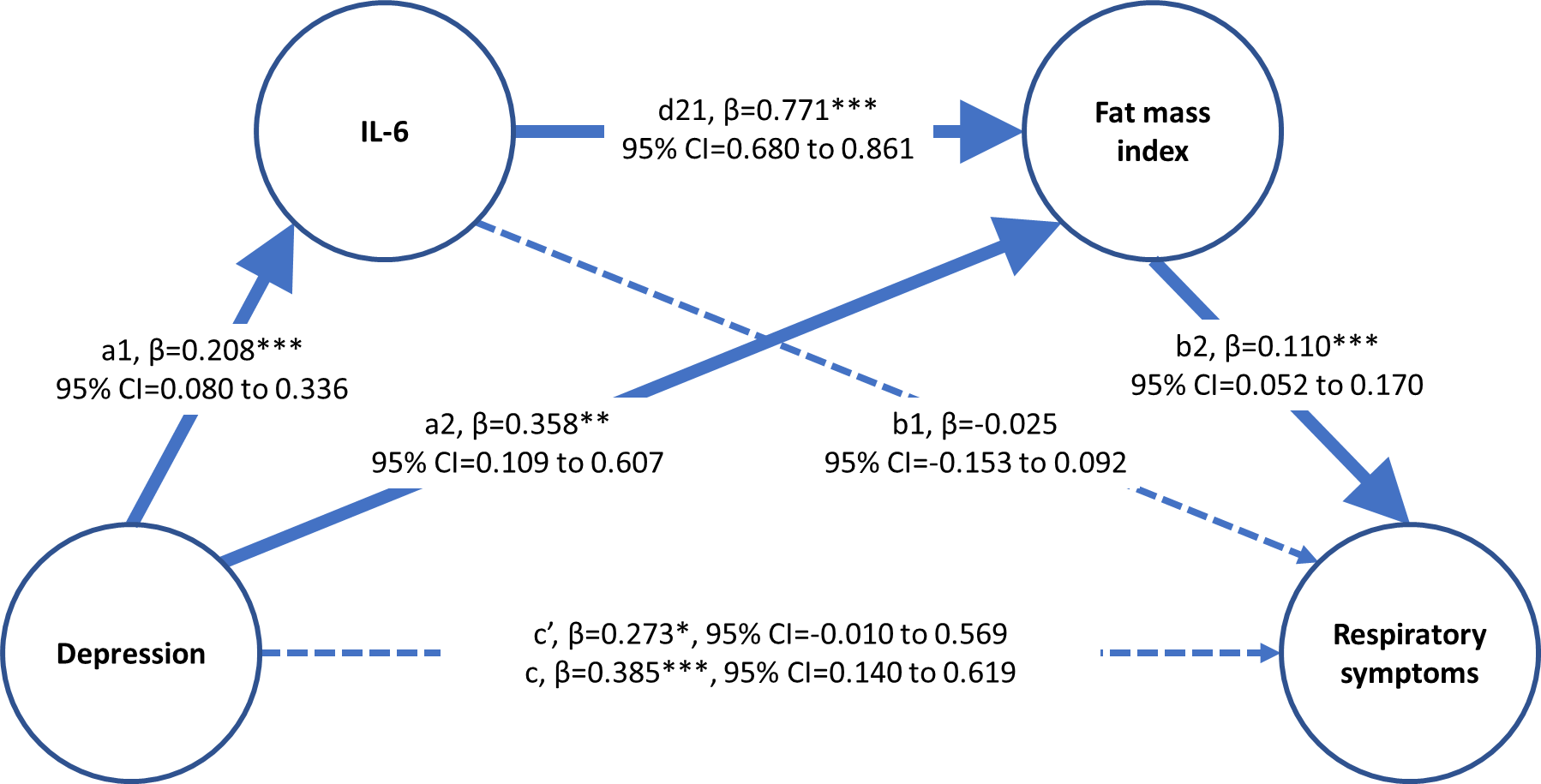


**eFigure 3. Serial mediation model linking IL-6 and respiratory symptoms (n=1742).** Effects were estimated using Bayesian based methods and presented as posterior mean with percentile-based 95% Bayesian credible interval of β. The indirect effect (such as a1*b1, a2*b2, and a1*d21*b2) and the ratio of indirect to total effect is presented in eTable 6.

*: the proportion of Bayesian credible interval less than 0 (Bayesian p value) <0.05; **: p<0.01; ***: p<0.001.
